# Tian Wang Bu Xin Dan for Insomnia: A Systematic Review of Efficacy and Safety

**DOI:** 10.1155/2019/4260801

**Published:** 2019-03-24

**Authors:** Xi-qian Yang, Ling Liu, Shu-ping Ming, Jie Fang, Dong-nan Wu

**Affiliations:** ^1^The TCM Clinical Institute, Hubei University of Chinese Medicine, Hubei 430065, China; ^2^The First Clinical Institute, Hubei University of Chinese Medicine, Hubei 430065, China; ^3^Encephalopathy Department, Hubei Provincial Hospital of TCM, Hubei 430061, China

## Abstract

**Background:**

The Traditional Chinese Medicine (TCM) Tian Wang Bu Xin Dan (TWBXD) has been used widely for treating insomnia in China. The purpose of this meta-analysis was to evaluate the efficacy and safety of TWBXD in the treatment of insomnia.

**Objective:**

This study evaluated the efficacy and safety of TWBXD for insomnia.

**Methods:**

We searched seven main databases including PubMed, EMBASE, Cochrane Central Register of Controlled Trials, Chinese Biomedicine Database (CBM), China National Knowledge Infrastructure (CNKI), Chinese Scientific Journals Database (VIP), and Wan-fang. We identified randomized-controlled trials (RCTs) for insomnia treatment involving TWBXD, TWBXD combined with conventional Western medicine, and conventional Western medicine from their inception to May 2018. The quality of literature was evaluated by Cochrane assessing tool to reduce the risk of bias. Meta-analysis and heterogeneity of results across the trials were performed. RevMan 5.3 was used to synthesize the results.

**Results:**

14 studies involving 1,256 participants were identified in this systematic review. Methodological deficiencies existed in most of the included trials. Few studies described the generation of a random sequence in detail, the concealment of allocation, and the methods of blinding. No placebo was used in treatment. 12 trials compared TWBXD with conventional Western medicine and 2 trials compared TWBXD combined with conventional Western medicine. The results of our meta-analysis showed relative benefits in effective rates in favor of TWBXD (Odds Ratio [OR] 2.71, 95% confidence interval [CI] 1.67 to 4.39, P < 0.00001) and TWBXD combined with conventional Western medicine (OR 5.05, 95% CI 1.58 to 16.12, P=0.006). The Pittsburgh Sleep Quality Index (PSQI) scores showed similar results, which favored TWBXD (Weighted Mean Difference [WMD] -1.82, 95% CI -3.00 to -0.64, P=0.003). Only 5 trials reported adverse events, whereas the other 9 trials did not provide the safety information.

**Conclusion:**

This review demonstrates that although the effects of TWBXD on insomnia were promising, they need to be interpreted with caution, due to the poor methodological quality and the small number of trials of the included studies. TWBXD seems to be generally safe, but there is insufficient evidence to make conclusions on the safety because fewer studies reported the adverse events. Further studies on a larger scale with more rigorous designs are required to evaluate the role of TWBXD in the insomnia treatment.

## 1. Introduction

Insomnia refers to a subjective experience in which patients are not satisfied with sleep time and quality and affect social function during the day, mainly manifested as difficulty falling asleep, sleep maintenance disorder, early awakening, poor sleep quality, and less total sleep time [1].

Current therapeutic strategies mainly include benzodiazepine receptor agonists, melatonin receptor agonists, and hypnotic antidepressants. However, the current routine pharmacotherapy is less than satisfactory, such that the treatments have many issues, including contraindications, side effects, high costs, and addictions [2]. Recently, the use of complementary and alternative medicine (CAM), such as the Tian Wang Bu Xin Dan, is increasing due to their curative effects, low cost, and wide-range of applications.

Treatment with TWBXD has achieved promising clinical effects in terms of better clinical efficacy and fewer adverse effects. However, the strength of this conclusion is limited by the small sample sizes of most trials. In addition, no previous systematic review or meta-analysis has evaluated the effect of TWBXD for treating insomnia.

In this study, we systematically evaluated the efficacy and safety of TWBXD in insomnia based on available randomized-controlled trials (RCTs).

## 2. Methods

### 2.1. Search Strategy

Systematic searches were conducted with the terms “Sleep Initiation and Maintenance Disorders” or “Early Awakening” or “Insomnia” or “Tianwang Buxin” or “Sleep Initiation Dysfunction” using the following databases: PubMed, EMBASE, Cochrane Central Register of Controlled Trials, Chinese Biomedicine Database (CBM), China National Knowledge Infrastructure (CNKI), Chinese Scientific Journals Database (VIP), and Wan-fang through May 2018. We identified relevant controlled trials on Insomnia, Sleeplessness, or CI using the Cochrane Controlled Trials Register. Other sources searched were conference proceedings, abstracts, thesis dissertations, poster presentations, and materials from professional society meetings.

### 2.2. Selection Criteria

Included trials met the following criteria: (1) Parallel-group RCTs, irrespective of blinding or publication status. Crossover trials were only included for the first phase data. Quasi-randomized trials were excluded. (2) Patients diagnosed with insomnia according to internationally accepted diagnostic criteria for insomnia, including the International Classification of Diseases (ICD-10) [3], the Diagnostic and Statistical Manual of Mental Disorders (DSM-IV and DSM-IVR) [4, 5], Chinese Classification of Mental Disorders (CCMD-3) [6]. (3) More than 30 participants per group in included trails.

### 2.3. Types of Outcome Measures

The primary outcome measurement was sleep questionnaires such as the Pittsburgh Sleep Quality Index (PSQI) [7]. The secondary outcome measurement was the clinical effective rate based on response evaluation criteria in TCM treatment of insomnia [8] and the adverse events. In Guideline for Clinical Trials of New Patent Chinese Medicines, evaluation standards for clinical therapeutic effects were as follows [8]: (1) clinical cure: sleep time to restore normal sleep time or the nighttime sleep duration of more than 6 hours, deep sleep, and full of energy after waking up; (2) markedly effective: significant improvement of insomnia; sleep time increased over 3 hours compared with the previous sleep time; an increase of the depth of sleep; (3) effective: amelioration in symptoms; sleep time increased less than 3 hours compared with the previous sleep time; (4) ineffective: no significant improvement of insomnia or deteriorated after treatment.

### 2.4. Data Extraction and Quality Assessment

Two reviewers independently extracted the data from the selected trials into a standard data extract form. The extracted data included age, gender, trial duration, treatment, outcome, and adverse reactions. We evaluated the methodological quality of the included trials in accordance with the Cochrane risk of bias tool [9]. The judgment of risk of bias includes random sequence generation, allocation concealment, blinding of participants and personnel, blinding of outcome assessments, incomplete outcome data, selective reporting, and other sources of bias. Any disagreements were resolved by discussion with a third reviewer.

### 2.5. Statistical Analysis

The pooled summary was expressed as odds risk (OR) with 95% confidence interval (CI) for discontinuous variables. Heterogeneity across trials was tested using the I^2^ statistic and Cochrane Q statistic. If I^2^>50% and p>0.10 for the Cochrane Q statistic, we selected a random effect model; otherwise, a fixed effect model was applied. Subgroup analysis was performed based on the different treatment durations. To assess potential publication bias, a funnel plot was carried generated. All statistical analyses were conducted using Review Manage software 5.3 (Cochrane Collaboration, Oxford, UK).

## 3. Results

### 3.1. Description of Studies

**Figure 1:**
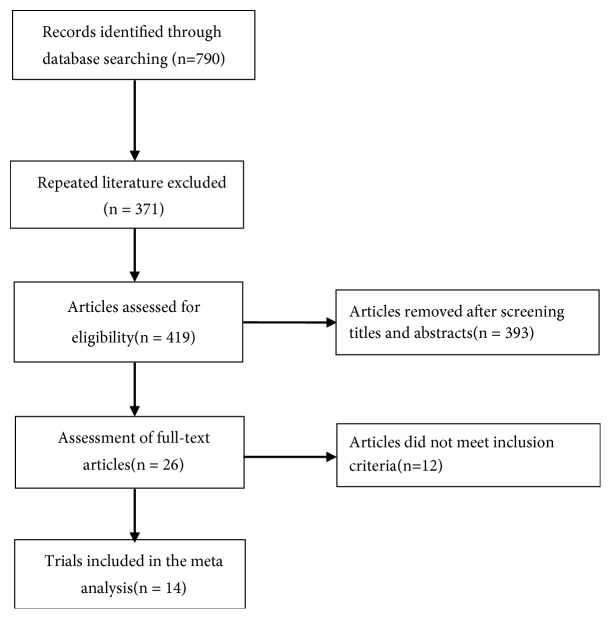
Flowchart of trial selection process.

A total of 790 records were retrieved from the above-mentioned databases and through the manual literature search. Of these, 371 articles were excluded upon exclusion of duplicated publications. After reading the titles and abstracts, another 393 articles were removed. Thus, 26 full-text articles were assessed for eligibility. After a detailed assessment of the full-text papers, an additional 12 articles were excluded mainly because they did not satisfy our predefined inclusion criteria. Finally, 14 RCTs [10–23] were included in the meta-analysis (Figure 1).

### 3.2. Trial Characteristics

Table 1 presents the baseline characteristics of patients in the trials included in the meta-analysis. All the included trials were carried out in China and published from 2006 to 2018. The sample sizes ranged from 60 to 133. A total of 1,256 patients were included in the 14 trials. The TWBXD group consisted of 655 patients, while the conventional Western medicine (WM) treatment group consisted of 601 patients. 11 trials were diagnosed according to CCMD [10–14, 16, 19–23], 2 were based on ICD-10 [15, 17], and 1 was diagnosed according to TCM syndrome diagnosis and treatment standard. All trials adopted TWBXD monotherapy or adjunct therapy in the treatment group for insomnia. Among the 14 trails, 12 were monotherapy [10–18, 21–23] and the rest of 2 studies [19, 20] were combined with the WM to treat the insomnia. The duration of treatment was varied from 2 weeks to 8 weeks. Clinical efficacy was observed in 13 studies [10–16, 18–23]; PSQI score was tested in 6 studies [11, 12, 15, 17, 21, 22]. Adverse effects were reported in 6 studies [11, 15, 21–23], while there is no mention in the other studies (Table 1).

### 3.3. Methodological Quality of the Included Trials

**Figure 2:**
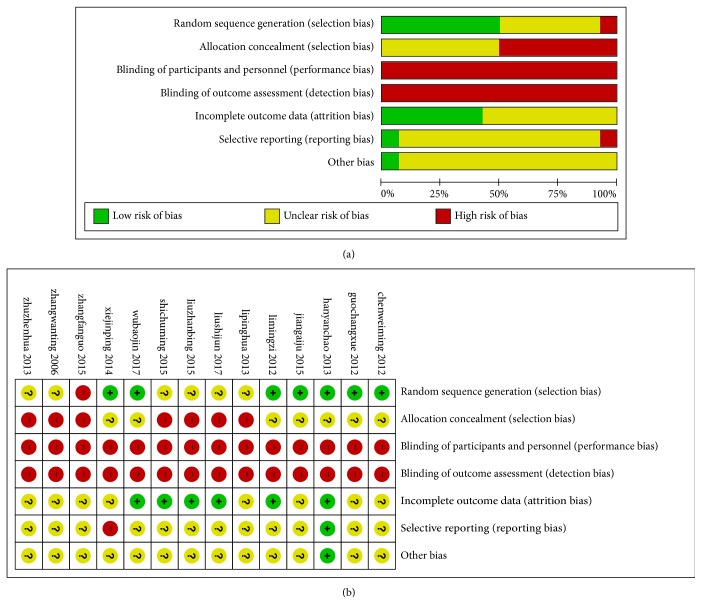
Graph of bias risk (a) and summary of bias risk (b).

Figure 2 summarizes the methodological quality of the 14 RCTs. 6 trials [11, 13, 15, 17, 18, 23] were randomized using random number tables to generate a sequence (appropriate), 1 trials [19] used a temporal sequence for randomization (inappropriate), and the remaining trials only mentioned randomization without detailed methods. 1 trial [15] reported the patients' reasons for withdrawal or loss to follow-up.

### 3.4. The Primary Outcome

**Figure 3:**
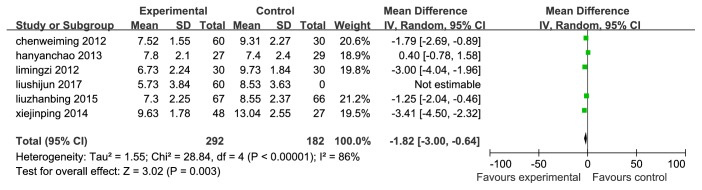
Forest plots based on the PSQI total score.

We use the PSQI as primary outcome, and there were 6 RCTs [11, 12, 15, 17, 21, 22] comparing TWBXD monotherapy with conventional medicine. As shown in Figure 3, significant heterogeneity (I^2^=86%, p<0.001) between trials was observed, and thus, we selected a random effect model. Overall, TWBXD group could reduce PSQI total score better compared with WM treatment alone (WMD=-1.82, 95% CI [-3.00,-0.64], P=0.003). We use the methods of eliminating each test one by one to analyze the sources of heterogeneity, showing that the heterogeneity between the trials is still significant (I^2^>50%), but the result is stable (Figure 3).

**Figure 4:**
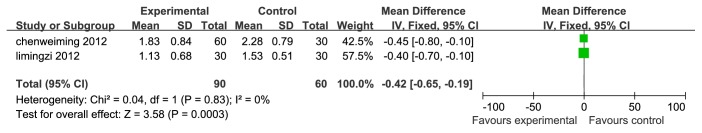
Forest plots based on the time required to fall asleep.

**Figure 5:**
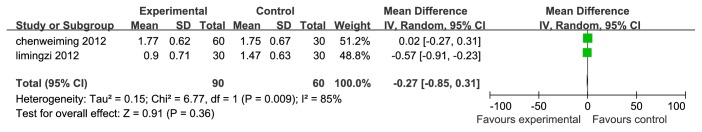
Forest plots based on the quality of sleep.

2 trials [11, 12] analyze the time required to fall asleep and the quality of sleep. The result indicates that the effect of TWBXD is better than that of WM in shortening the time of falling asleep (WMD=-0.42, 95% CI [-0.65,-0.19], P=0.0003) and there was no difference in the quality of sleep between this two groups (WMD=-0.27, 95% CI [-0.85,0.31], P=0.36) (Figures 4 and 5).

### 3.5. The Secondary Outcome

**Figure 6:**
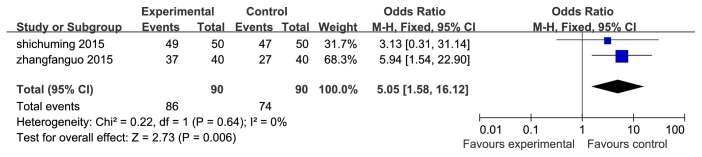
Meta-analysis of clinical effective rate of TWBXD with WM versus WM.

**Figure 7:**
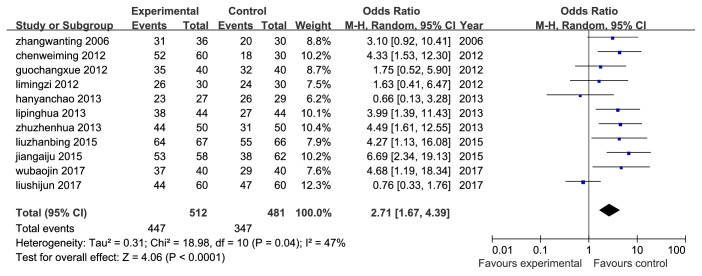
Meta-analysis of clinical effective rate of TWBXD monotherapy versus WM.

13 RCTs were assessed by clinical effective rates. Among the 13 trails, 11 [10–16, 18, 21–23] were comparing TWBXD monotherapy with conventional medicine, and the rest 2 studies [19, 20] were combined with the WM. The results showed a significant difference in favor of TWBXD with or without WM (OR=5.05, 95% CI [1.58,16.12], P=0.006; OR=2.71, 95% CI [1.67,4.39], P<0.0001) (Figures 6 and 7).

Adverse event monitoring was only reported in 5 studies [11, 20–23]. No serious adverse effects were mentioned in these studies. Li et al. [11] reported that there was no adverse event happened in the TWBXD group or WM group. In the study by Si et al., Liu et al., and Wu et al. [20, 22, 23], no adverse event happened in the TWBXD with or without WM group; there were 16 cases occurred in the WM group. Among the adverse events, six patients had the headache, one patient had erythra, and the nine patients had thirst. Liu et al. [21] reported that 2 events occurred in TWBXD group, and 9 events happened in the WM group.

## 4. Discussion

### 4.1. Summary of Evidence

To the best of our knowledge, this is the first systematic review and meta-analysis to evaluate the efficacy and safety of TWBXD for the treatment of insomnia. In this review, we analyzed treatment effects in 1,256 patients with insomnia from 14 trials [10–23]. Meta-analyses of these RCTs over the course of 2-8 weeks show that TWBXD seems to be beneficial for reducing the time to fall asleep. These clinical benefits are mainly associated with TWBXD. The safety of TWBXD treatment was also examined. There were only 1 case with minor adverse events in the TWBXD or TWBXD combined with WM group, which is in agreement with long-term clinical experience. However, the potential benefits of TWBXD in the treatment of insomnia remain uncertain due to methodological deficiencies and limited sample size for inclusion in the study. Therefore, recommendations for clinical practice should be cautious.

### 4.2. Limitations of the Review

First, some methodological deficiencies existed in most of the included trials. Few studies described the generation of a random sequence in detail, the concealment of allocation, and the methods of blinding. Hence, selection bias may exist in our analysis. No placebo was used in the treatment as well, which reduced the possibility of blinding and thus increased the possibility of detection bias or performance bias in the implementation process of the trial, as well as reporting bias in outcome evaluation [24, 25]. Dropouts also were not mentioned in all studies. We attempted to contact the authors via telephone or e-mail to obtain additional statistical data or methodological information, but most of the detailed responses could not be obtained. In addition, there were only 5 studies [11, 20–23] that reported adverse events in this systematic review, and the incidences of treatment-effect adverse events were used as the judgment of safety, which could not be considered as an objective indicator for severity evaluation of adverse reactions. A side effect scale, such as TESS, should be adopted and standardized as the safety evaluation method. In addition, no trials conducted pretrial estimation of sample size, which indicated the lack of statistical power to ensure appropriate estimation of the therapeutic effect [26].Thus, specific evidence for efficacy and safety are required in future studies.

Secondly, substantial heterogeneity was observed in the pooled total effective rate. The reason for the heterogeneity may be correlated with the use of different types of WM [27] and different doses of the TWBXD.

Third, publication bias may be a serious problem in reported herbal trials. Current studies show that Asian countries, including China, typically report a large proportion of positive results, although these studies were independently selected by the two authors and are strictly based on inclusion and exclusion criteria [24]. No negative results were found in this evaluation [28–31]. All of these studies were conducted and published in China, and most of the studies showed that TWBXD performed better than the WM group. A possible reason for these negative results could be explained by selection bias in the process of participant recruitment [32].

## 5. Conclusion

There is insufficient evidence regarding the efficacy of TWBXD for the treatment of insomnia because of the poor methodological quality and the small number of trials of the included trials. TWBXD is generally safe, but there is insufficient evidence to make conclusions on the safety because fewer studies reported the adverse events. More high-quality RCTs with large sample sizes are needed to verify the current findings.

## Figures and Tables

**Table 1:** Basic characteristics of the included studies.

Studies	Sample		Interventions		Duration (weeks)	Outcome index	Adverse effects	
	Trial	Control	Trial	Control			Trial	Control
Zhangwanting 2006	36	30	TWBXD	Estazolam	2	①	-	
Limingzi 2012	30	30	TWBXD	Bailemian Capsule	4	①②③④	0	0
Chenweiming 2012	60	30	TWBXD	Estazolam	8	①②③④	-	
Guochangxue 2012	40	40	TWBXD	Estazolam	2	①	-	
Lipinghua 2013	44	44	TWBXD	Estazolam + oryzanol	4	①	-	
Hanyanchao 2013	32	32	TWBXD	Alprazolam	4	①②	-	
Zhuzhenhua 2013	50	50	TWBXD	Estazolam + oryzanol	4	①	-	
Xiejinping 2014	48	27	TWBXD	Diazepam	4	②	-	
Jiangaiju 2015	58	62	TWBXD	Estazolam	4	①	-	
Zhangfanguo 2015	40	40	TWBXD + Diazepam	Diazepam	4	①	-	
Shichuming 2015	50	50	TWBXD + Estazolam	Estazolam	4	①	0	10
Liuzhanbing 2015	67	66	TWBXD	Alprazolam	4	①②	2	9
Wubaojin 2017	40	40	TWBXD	Estazolam +oryzanol	4	①	0	1
Liushijun 2017	60	60	TWBXD	Estazolam	4	①②③④	0	5

① Clinical effective rate; ② PSQI total score; ③ time required to fall asleep; ④ Quality of sleep.
